# Constructing network topologies for multiple signal-encoding functions

**DOI:** 10.1186/s12918-018-0676-5

**Published:** 2019-01-11

**Authors:** Lili Wu, Hongli Wang, Qi Ouyang

**Affiliations:** 1 The State Key Laboratory for Artificial Microstructures and Mesoscopic Physics, School of Physics, Peking University, Beijing, 100871 China; 2 Center for Quantitative Biology, Peking University, Beijing, 100871 China; 3 Peking-Tsinghua Center for Life Sciences, Peking University, Beijing, 100871 China

**Keywords:** Enzymatic networks, Signal-encoding, Network motifs, Design principle

## Abstract

**Background:**

Cells use signaling protein networks to sense their environment and mediate specific responses. Information about environmental stress is usually encoded in the dynamics of the signaling molecules, and qualitatively distinct dynamics of the same signaling molecule can lead to dramatically different cell fates. Exploring the design principles of networks with multiple signal-encoding functions is important for understanding how distinct dynamic patterns are shaped and integrated by real cellular networks, and for building cells with targeted sensing–response functions via synthetic biology.

**Results:**

In this paper, we investigate multi-node enzymatic regulatory networks with three signal-encoding functions, i.e., dynamic responses of oscillation, transient activation, and sustained activation upon step stimulation by three different inducers, respectively. Taking into account competition effects of the substrates for the same enzyme in the enzymatic reactions, we searched for robust subnetworks for each signal-encoding function by three-node-network enumeration and then integrated the three subnetworks together via node-merging. The obtained tri-functional networks consisted of four to six nodes, and the core structures of these networks were hybrids of the motifs for the subfunctions.

**Conclusions:**

The simplest but relatively robust tri-functional networks demonstrated that the three functions were compatible within a simple negative feedback loop. Depending on the network structure, the competition effects of the substrates for the same enzyme within the networks could promote or hamper the target functions, and can create implicit functional motifs. Overall, the networks we obtained could in principle be synthesized to construct dynamic control circuits with multiple target functions.

**Electronic supplementary material:**

The online version of this article (10.1186/s12918-018-0676-5) contains supplementary material, which is available to authorized users.

## Background

The relationship between structure and function in biological circuits is a focus of systems biology [1, 2]. Structure reflects the topology of interactions within a circuit, and function is usually considered a static quantity (a steady state) or a temporal behavior (dynamics) of the circuit’s output [3]. In various signaling systems, cells transmit information via dynamic control of signaling molecules [3]. The information about the identity and quality of a stimulus can be encoded in temporal patterns of a signaling molecule by modulating its amplitude, duration, frequency and so on. Cells mediate gene expression programs and induce cellular responses according to the sequential dynamics of signaling molecules [3–13]. Furthermore, signaling dynamics usually have greater information transmission capacities compared to non-dynamics responses [14–16]. For example, the tumor suppressor p53 shows a series of pulses with number modulation under γ-radiation, resulting in cell-cycle arrest. By contrast, UV radiation triggers a single p53 pulse with a dose-dependent amplitude and duration, leading to apoptosis [3, 17, 18]. In yeast, the transcription factor Msn2 plays a major role in the general stress response. Glucose limitation stress induces nucleocytoplasmic shuttling of Msn2. The duration of the initial peak and frequency of the sequential bursts in Msn2 increase with the strength of the stress [6, 19, 20]. Under osmotic stress, Msn2 exhibits a similarly initial nuclear peak and returns to normal. The duration of the peak increases with the osmotic pressure [3, 21–25]. In addition, oxidative stress leads to prolonged nuclear Msn2 accumulation whose amplitude is dependent on the concentration of H_2_O_2_ [6, 26, 27]. Other systems, such as ERK, NF-κB and Crz all drive distinct responses via dynamic control [3, 28–30].

These observations in real signaling systems raise fascinating questions about how distinct input information is encoded into different dynamic patterns of the signaling molecule and the design principles for such networks with multiple signal-encoding functions. Instead of considering the complex molecular interaction details in real signaling networks, we here intend to perform a theoretical investigation of multi-node enzymatic regulatory networks with three signal-encoding functions, i.e., dynamic responses of oscillation, transient activation, and sustained activation when a respective input node is stimulated. These dynamic patterns are similar to the observed dynamic response of Msn2 in yeast under glucose limitation, osmotic stress, and oxidative stress, respectively.

General computational search strategies for detecting network topologies for a target function include evolutionary algorithms and the network enumeration approach [2]. These approaches have been used to explore simple network solutions for dynamic functions such as oscillations [31–33], switch-like responses [34, 35], adaptation [36–38] and dose-response alignment [39]. Core motifs and design principles for these individual dynamic functions have been investigated previously. By contrast, multi-functional networks that can execute several distinct functions have rarely been considered [1], despite the accumulation of experimental observations of multiple signal-encoding processes. The stumbling blocks for searching multi-functional networks are mainly due to the increased network scale and the functional complexity, which require enormous computing power. As the multi-functional networks we consider have at least four nodes, with three nodes for inputting three external signals and one node for output, it is practically infeasible to enumerate all possibilities to detect multi-functional network topologies. Evolutionary approaches are also inapplicable for deducing the architectural landscape of tri-functional networks. Accordingly, here we adopt the scenario of module combination for searching multi-functional networks capable of the desired multiple signal-encoding function. The module combination approach has recently been used to generate bi-functional networks by hybridizing simpler mono-functional modules [1]. It was also applied successfully to detect network structures with Pavlovian-like functions capable of learning and recalling [40].

In our calculations, the external stimulus, which is a temporal step function, acts on three different input nodes, and generates on the output node sustained oscillations (stable limit cycle), transient activation (adaptation), and sustained activation, respectively. This mimics the signal-encoding of Msn2 in yeast in response to glucose limitation, osmotic stress, and oxidative stress, respectively. To obtain the target multi-functional networks, we search first for robust subnetworks (sub-functional modules) for each target input-output response by enumerating all three-node network topologies and then meld different modules through node merging. All computations are performed with enzymatic regulatory networks. In addition, we take competition effects among different substrates into account in situations when two or more types of substrates bind competitively to the same enzyme. This ubiquitous hidden interaction has been considered in gene regulatory networks [41], but is commonly omitted in most theoretical or experimental models in enzymatic circuits [42]. We find that competition effects can increase the diversity of functional modules by creating implicit cross couplings within the circuits, whereas in combined networks, competition effects can promote or hamper the overall functionality. These findings may be helpful for understanding multi-functional networks in real biological systems and also provide a guide to construct information-encoding circuits in synthetic biology.

## Results

### Module selection for each signal-processing function

We use the method of three-node-network enumeration to explore the functional networks of oscillation (F_1_) and adaptation (F_2_), respectively. In a two- or three-node network, one node serves as the input node (Node R) and one as the output node (Node O), and a third node will be the intermediate node (Node M). For each topology, we sample 10,000 parameter sets using the Latin hypercube sampling method [43]. For each parameter set, the ordinary differential equations that describe the network dynamics in response to one of the two stimuli are then solved numerically. The topologies with at least one or more parameter sets that produce the targeted dynamical function are considered functional circuits. The robustness of sub-functions F_1_ and F_2_ is measured by the number of parameter sets, i.e., Q_1_- and Q_2_-values, that generate the target dynamic functions. For functions F_1_ and F_2_, we select the network topologies with relatively high Q-values and fewer edges as functional F_1_ and F_2_ module pools.

The oscillation (F_1_) pool contains 73 networks with 3 to 6 edges with Q_1_ > 50 and 8 simplest networks with 3 edges with 10 < Q_1_ < 50 (as marked in Fig. 2a). Clustering analysis of these networks (Fig. 2b) shows that the basic oscillation motifs for achieving function F_1_ are negative feedback loops between the three nodes. Motifs a_1_ and a_3_ are simply two forms of the repressilator, and motifs a_2_ and a_4_ are the delayed feedback loop. All 73 networks contain explicitly one of the two negative feedback loops which are well-known motifs for oscillations. This is true even for networks with edge numbers greater than 6. Additionally, the F_1_ functional pool also contains the 8 simplest networks. They do not explicitly have the structure of a repressilator or delayed feedback loop (Fig. 2c). These topologies achieve self-sustained oscillations because one of the two basic motifs of the repressilator and delayed feedback loop is implicitly contained in their topologies due to the hidden interactions. Two such examples are illustrated in Fig. 2g, where the oscillations are achieved through the implicit a_3_ and a_4_ motifs. Thus, the modular pool of F_1_ consists of 81 networks as marked in Fig. 2a.

Similarly, as marked in Fig. 2d, the F_2_ functional pool also consists of 81 selected networks, i.e., 76 networks with 3 to 6 edges and Q_2_ > 55, and 5 networks with 3 edges and 10 < Q_2_ < 55. Clustering analysis of the 76 networks reveals the backbones of the functional circuits (Fig. 2e), two negative feedback loops with a buffer node (motifs b_1_, b_3_), a delayed feedback loop (motif b_2_), and an incoherent feedforward loop with a proportional node (motif b_4_). The motifs for function F_2_ are similar to Ma’s results [31], but different in that motifs b_3_ and b_4_ (for which Q_2_ < 55) are less robust than motifs b_1_ and b_2_ (for which Q_2_ > 55). This difference is attributable to the inclusion of competitive regulations in both motifs b_3_ and b_4_: node M_3_ and node O_3_ compete for enzyme R_3_ to convert from the inactive to active state. Consequently, there exit implicit positive interactions between nodes M_3_ and O_3_, and the regulatory interactions are more complex than those discussed in [36], in which the competitive effect was ommitted. Figure 2f illustrates the 5 networks with three nodes and lower Q_2_ value as marked in Fig. 2d. These simple functional circuits all have implicit interactions, of which two are motifs b_3_ and b_4_. The left three motifs have very different topologies from the ordinary adaptation motifs. Checking the implicit interactions reveals that these seemingly very different adaptation motifs all have implicitly the core structures discussed in detail in [36]. Two such examples are illustrated in Fig. 2h where adaptation is achieved through the core structures of b_1_ and b_3_.

**Fig. 1:**
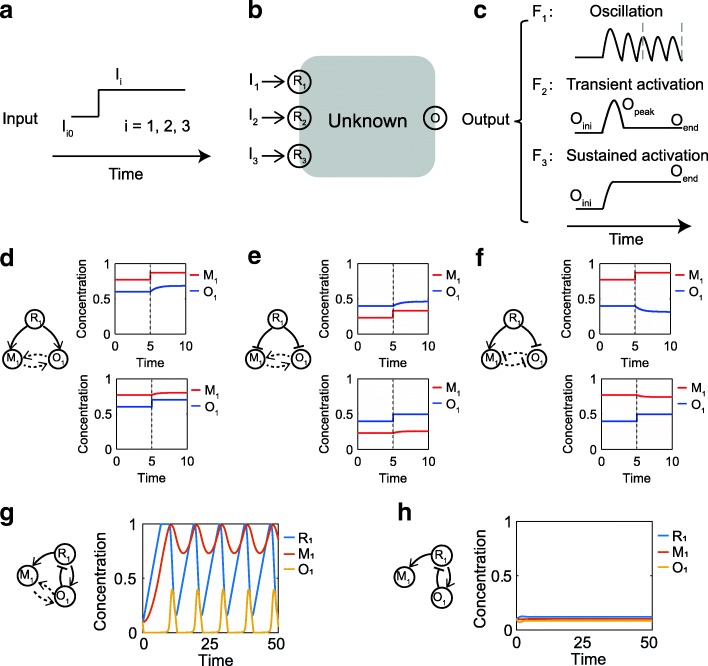
Defining objective functions for three distinct signal-encoding processes. **a** The temporal step functions (jump from *I*_i0_ = 0.1 to *I*_i_ ∈[0.5, 1], *i* = 1,2,3) represent three external signals that mimic glucose limitation, osmotic stress or oxide stress in the yeast Msn2 stress responses, respectively. **b** The signals are received by the receptor nodes *R*_1_, *R*_2_, and *R*_3_, respectively, one signal at a time and are respectively encoded into different dynamics of the circuit’s output node *O* through the unknown signaling network. **c** The target output dynamics of the output node upon stimuli, i.e., F_1_ for oscillation (with Period > 0.1, Amplitude > 0.1), F_2_ for transient activation (with |O_end_ – O_ini_| < 0.1, O_peak_ – O_ini_ > 0.2, |O_end_ – O_ini_| < 0.5(O_peak_ – O_ini_)), and F_3_ for sustained activation (O_end_ – O_ini_ > 0.2), respectively. (d-f) Illustrations of implicit interactions between M_1_ and O_1_ when active enzyme R^*^_1_ simultaneously activates (**d**), or deactivates (**e**) the substrate enzymes M_1_ and O_1_, or when it activates M_1_ and deactivates O_1_ (f). The dashed links in the circuits represent the implicit interactions of mutual activations (d,e) and deactivations (**f**), respectively. (g,h) demonstrates an example where the same circuit exhibits completely distinct dynamical behaviors of oscillations (**g**) and steady state (**h**) when the substrate competition is considered (**g**) or not (**h**). Refer to additional file 1 for the rate equations and parameter values used in (d-h)

**Fig. 2:**
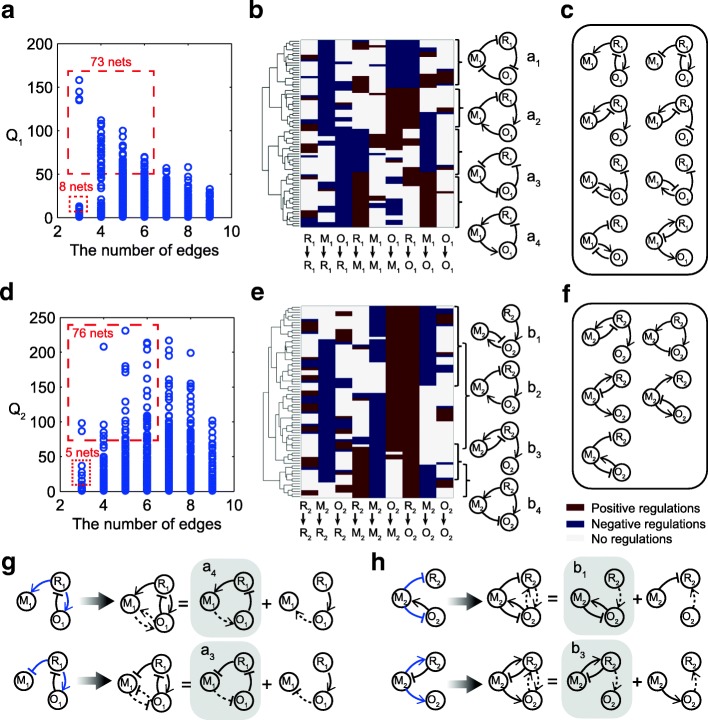
Modular pools for oscillation and adaptation. **a** Distribution of networks capable of achieving function F_1_ in the space of the Q_1_-value and the number of edges. A total of 81 three-node networks (73 plus 8) are selected into the F_1_ modular pool, as highlighted in the dashed boxes. **b** Clustering of 73 networks as marked in (a). Each row in the clustering figure demonstrates the regulations among the three nodes within a network. Positive, negative and null regulations within the networks are denoted by red, green and black, respectively. The core oscillation motifs are labeled a_1_, a_2_, a_3_ and a_4_ as shown. **c** Eight simple F_1_ circuits, as marked in (a), that contain explicitly no oscillation motifs. **d** ~**f** Similar analyses of 81 three-node networks (76 plus 5) that construct the F_2_ modular pool. (f) The 5 simplest topologies as marked in (d) all of which contain implicit interactions due to the competitive effects. (g, h) Examples of functional networks with implicit motifs for functions F_1_ and F_2_, respectively, with competitive edges highlighted in blue. The dashed linkages denote the implicit interactions due to competition effects. The implicit regulations plus explicit linkages in the network form the ordinary F_1_ and F_2_ motifs (highlighted in grey)

As demonstrated in Fig. 1 and Fig. 2, the inclusion of competition effects into the circuit dynamical description generates mutual activation or inhibition interactions. Taking these implicit interactions into account benefits analysis of the functioning of networks when we judge whether a specific dynamical behavior is possibly supported in a network topology. It has been well known that oscillations are generated in circuits with topologies of negative feedback loops and that adaptation is typically achieved with topologies of incoherent feedforward or negative feedback loop with buffering node. For the circuits that do not have apparent topologies that support oscillations or adaptations, one can add the implicit interactions onto the network and then check whether the newly formed network have the core structure of oscillation or adaption. In this way, implicit functional motifs that are previously unknown can be found as depicted in Fig. 2g and Fig. 2h.

The functional circuits with sustained activation (F_3_) are not searched by enumeration. We prefer to adopt the simplest structure, i.e., a link of activation from the input node R_3_ to the output node O_3_. Thus, we have constructed the module pools for the sub-functions of oscillation (F_1_, with 81 topologies), transient activation (F_2_, with 81 topologies), and sustained activation (F_3_, with 1 topology), respectively.

### Module combination for bi-functional networks of F_1_ and F_2_

**Fig. 3:**
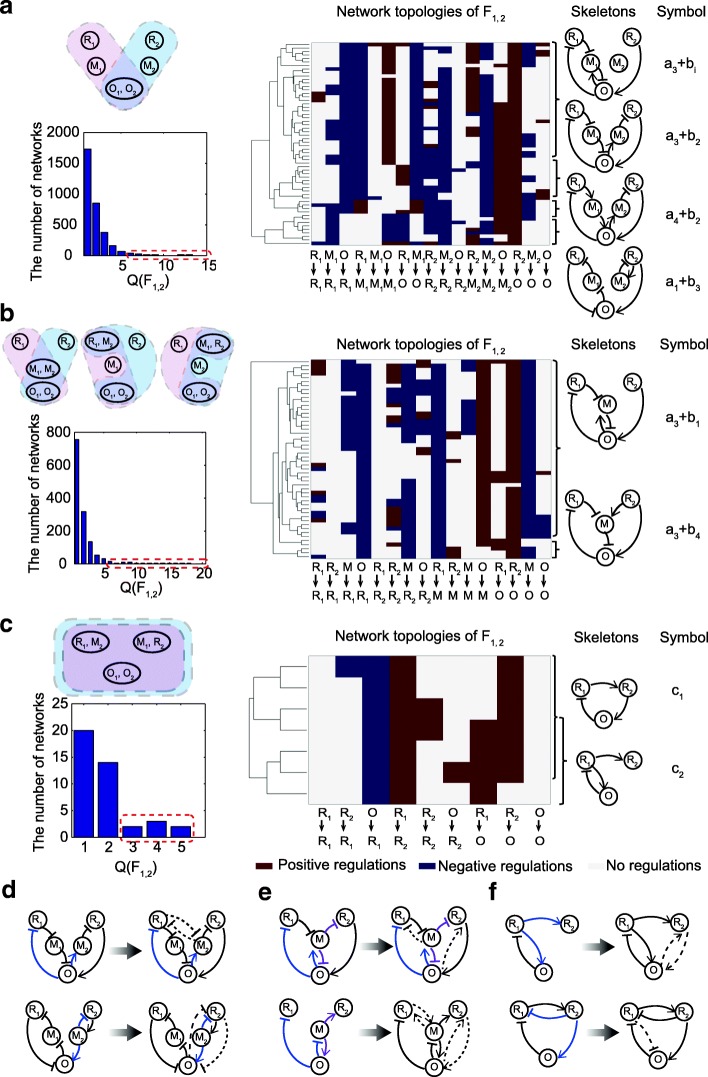
Bi-functional networks via module combinations. (**a**), (**b**) and (**c**) depict the results for one-node, two-node, and three-node merging, respectively. In each subfigure of (a), (b), (c), the upper left panel shows the form of node merging; two nodes in an ellipse represent the nodes that are merged, and the bottom left panel is the Q_1,2_ distribution of the combined bi-functional networks. Clustering analyses of the networks with relatively high Q_1,2_-values as marked by dashed red box in the histogram are depicted in the middle. The structure skeletons are illustrated on the right. **d**-**f** Examples of bi-functional combined networks that contain implicit interactions due to the competitive effect. Competitive edges are indicated in blue or purple. The dashed linkages represent the implicit interactions

By combining sub-function networks in the F_1_ and F_2_ module pools, we next construct F_1_-F_2_ bi-functional networks that encode the external signal received by nodes R_1_ and R_2_ respectively into oscillations and adaptation in the output node O. The network topologies from the two sub-functional pools are united by merging their nodes. As demonstrated in Fig. 3, we adopt the following node-merging rules:

(i). Output O nodes have to be merged with each other as we assume a unique output node.
(ii). M nodes may be merged with each other (unless there are contradictory linkages).
(iii). R nodes may be merged with M nodes from the other pathway (unless there are contradictory linkages).
(iv). R nodes for receiving signals cannot be merged with each other.
(v). R nodes are not permitted to merge with O nodes.

Here we do not consider the merging between R and O nodes and node merging within pathway. As there are two input nodes for receiving the signals and a unique output node in a bi-functional network, there are three possible ways to combine the F_1_ and F_2_ circuits as illustrated in the upper left of Fig. 3a-c. One approach is to simply merge the output nodes O_1_ and O_2_. Another is two-node merging of M_1_-M_2_, R_1_-M_2_, and R_2_-M_2_ in addition to the O_1_-O_2_ merging. The third approach is the three-node merging of O_1_-O_2_, R_1_-M_2_, and R_2_-M_1_.

We enumerate all possible networks combined from the two module pools as candidate topologies for bi-functional circuits. For each candidate network, we randomly sample 10,000 parameter sets and check whether it is bifunctional. The number of parameter sets that can achieve both oscillation and adaptation is recorded as the Q_1,2_-value to estimate the robustness of the bi-function. In the bi-functional networks with Q_1,2_ > 0, we obtain 3215 five-node, 1337 four-node, and 41 three-node networks by one-, two-, and three-node merging, respectively. The Q_1,2_ distributions of these three classes of bi-functional combined networks are illustrated in the lower left of Fig. 3a-c. The networks with relatively high Q_1,2_ are highlighted with red dashed boxes in the histograms of Fig. 3. These robust networks obtained by one-, two- and three-node merging are then clustered (middle panels in Fig. 3a-c). Structure skeletons from the clustering analyses are listed alongside (right panels in Fig. 3a-c). The robust bi-functional networks obtained via one-node and two-node merging are composed of hybrid motifs from the F_1_ and F_2_ module pools, such as hybrids of a_3_ + b_1_, a_3_ + b_2_, a_4_ + b_2_, and a_1_ + b_3_ in five-node combined networks and a_3_ + b_1_ and a_3_ + b_4_ in four-node combined networks. We find that the networks containing the hybrid motif a_3_ + b_1_ have the largest proportion; the mechanism for achieving the bi-function is given in Additional file 1. As three-node merging is full merging of the network, it is difficult to find very robust bi-functional networks with large Q_1,2_ values (no more than 5). Due to regulation conflicts, the F_1_ motifs (a_1_, a_2_, a_3_, and a_4_) and F_2_ motifs (b_1_, b_2_, b_3_, and b_4_) cannot be fully merged to form a bifunctional motif. Instead, the bi-functional networks of combination are derived from non-core motifs that can achieve both oscillation and adaptation, such as the structure skeletons c_1_ and c_2_ depicted in Fig. 3c.

By combining the nodes of functional F_1_ and F_2_ networks, the merged nodes function as a joint uniting the sub-functional networks. This results in competing effects between substrates and additional implicit interactions within the networks. This side effect of node merging could promote, hamper, or have little influence on the circuit’s function, depending on the strength of the competing strength. In Fig. 3d-f, we list several examples of bi-functional networks that contain implicit interactions as denoted by the dashed linkages. It is noted that the three-node bi-functional networks belong to the intersection of three-node networks in F1 and F2 pools, and that only a portion of networks in the intersection can achieve the bi-function. The node merging procedure does not discover new three-node networks that are bifunctional. For instance, all compatibly combined networks of oscillation motifs a_1_-a_4_ and adaptation motifs b_1_-b_4_ by full merging of the three nodes have a zero Q_1,2_ value.

### Extending the bi-functional networks into tri-functional networks

**Fig. 4:**
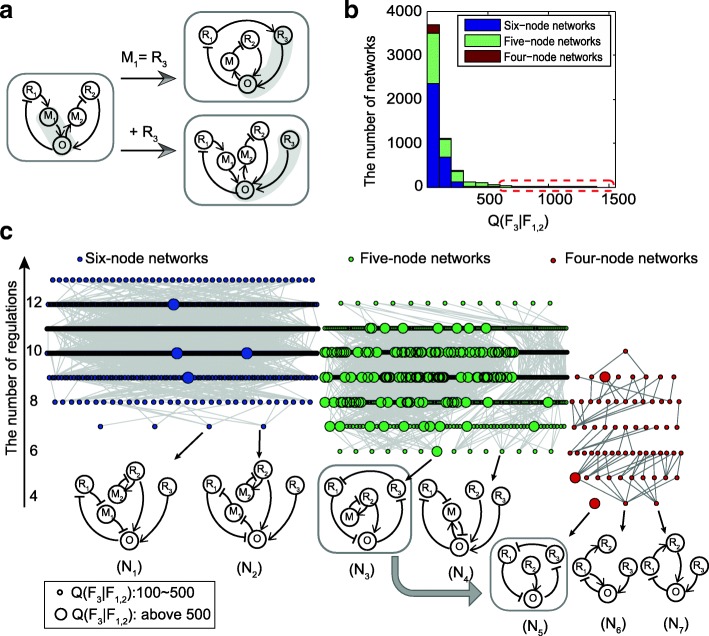
Tri-functional networks constructed from the bi-functional networks. **a** Two extension strategies for constructing tri-functional networks from bi-functional networks. A regulatory node that activates the output node O is adopted as the third input node (e.g., the node M_1_); otherwise, a new node R_3_ is added to receive the third signal. **b** The distribution of *Q*(*F*_3_| *F*_1, 2_) for all tri-functional networks obtained, with 141 tri-functional networks with *Q*(*F*_3_| *F*_1, 2_)> 500 (marked by dashed red box. **c** Structure diagram of all tri-functional networks for illustrating their structure relationships. The blue, green, and red colors represent six-, five- and four-node networks, respectively. Small and large dots represent tri-functional networks with *Q*(*F*_3_| *F*_1, 2_)> 100, and Q(*F*_3_| *F*_1, 2_)> 500, respectively. They are organized along the vertical axis with respect to the number of links in the network. Several network topologies with the least number of links are highlighted at the bottom

Next, we extend the bi-functional networks to the target tri-functional networks. As illustrated in Fig. 4a, a bi-functional network can be processed in two ways to include an extra node (R_3_) for receiving the third signal. One approach is to adopt an existing regulatory node (i.e., a node other than R_1_, R_2_ and O) as the receptor for input of the third signal. The other strategy is to introduce a new node as R_3_ into a bifunctional network for receiving the third signal. As the third function requires sustained activation of the output node O, an activation from node R_3_ to the output node O is simply added. For the circuit in Fig. 4a, the node M_1_ or M_2_ can be used as the third receptor node R_3_; otherwise, an additional node R_3_ is added that activates the output node. Both strategies add no new competitive regulations to the networks.

For each candidate tri-functional network obtained from the above extension strategies, we sample 1000 random parameter sets for the newly introduced parameters while keeping the parameters in the original circuits unchanged. The number of parameter sets that can achieve the target tri-functional behavior is recorded as the Q(F_3_|F_1,2_)-value, which manifests the robustness for the triple signal-encoding function (see Additional file 1). The distribution of *Q*(*F*_3_| *F*_1, 2_) for all tri-functional networks obtained is shown in Fig. 4b. There are 3190 tri-functional networks with six nodes, 2092 tri-functional networks with five nodes, and 255 tri-functional networks with four nodes. The networks with *Q*(*F*_3_| *F*_1, 2_) > 500 are highlighted in the red frame in Fig. 4b, with 4 six-node networks, 134 five-node networks, and 3 four-node networks.

**Fig. 5:**
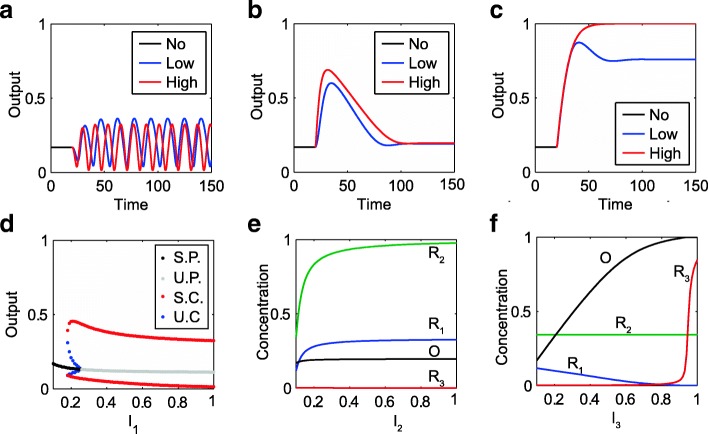
Dynamics analysis for the tri-functional network N_5_ in Fig.4c. **a** Oscillations result from I_1_ signal stimulation of two different strengths. **b** Transient activation or adaptation triggered by signal I_2_ of low and high strength. **c** Sustained activation when signal I_3_ of different amplitudes acts upon node R_3_. **d** Bifurcation diagram for function F_1_ when the amplitude of signal I_1_ is tuned. (**e**) and (**f**) show the steady state concentrations as functions of I_2_ and I_3_ signal strength, respectively. The low and high signal strengths in (a-c) correspond to 0.5 and 1.0, respectively. I_1_ = I_2_ = I_3_ = 0.1 are adopted when no signal is present. The unit of time is arbitrary. Other parameters used in simulations: *V*_1_ = 0.3156, *V*_2_ = 1.8362, *V*_3_ = 0.7503, *V*_4_ = 0.5559, *V*_5_ = 2.3514, *V*_6_ = 0.2716, *V*_7_ = 0.7010, *V*_8_ = 0.6726, *K*_1_ = 5.1160, *K*_2_ = 0.3030, *K*_3_ = 0.0111, *K*_4_ = 1.2833, *K*_5_ = 0.0259, *K*_6_ = 0.0277, *K*_7_ = 0.0014, *K*_8_ = 0.0099, *B* = 0.5

In Fig. 5c, we plot the structure diagram for all tri-functional networks with *Q*(*F*_3_| *F*_1, 2_) > 100 [44]. A dot denotes a tri-functional network, and networks with *Q*(*F*_3_| *F*_1, 2_) > 500 are represented by large dots. All networks are distributed with respect to the number of links and nodes in the networks. We have linked any two networks that could be transformed by the gain or removal of one regulatory interaction. Most of the tri-functional networks we obtained are connected, and complex tri-functional networks can evolve from simpler core topologies. Several core networks are illustrated at the bottom in Fig. 5c, i.e., the networks labeled from N_1_ to N_7_. Network N_4_ is constructed by uniting motifs a_3_ and b_1_ and adding the R_3_ node. The network N_3_ is devised on the bi-functional networks with hybrid motifs of a_1_ + b_3_ via the first strategy. It has two possible nodes (R_3_ and M) used as a buffer node for F_2_ and can be reduced to the network N_5_ by removing node M. The robustness of network N_5_ is comparable with that of network N_3_. Thus, network N_5_ is the simplest robust tri-functional network in our calculation. In the following section, we perform an analysis of the dynamics of network N_5_.

### Dynamics analysis for the simplest tri-functional network

For the simplest and functionally robust network N_5_ in Fig. 4c, we demonstrate its multiple signal-encoding dynamics in detail for illustrative purposes. The network dynamics in concentrations of active forms of enzymes R_1_, R_2_, R_3_ and O is described by the following ordinary differential equations,

1 $$ \left\{\begin{array}{c}\begin{array}{c}\frac{d{R}_1}{dt}={V}_1{I}_1\frac{1-{R}_1}{K_1+1-{R}_1}-{V}_5{R}_3\frac{R_1}{K_5+{R}_1}\\ {}\frac{d{R}_2}{dt}={V}_2{I}_2\frac{1-{R}_2}{K_2+1-{R}_2}-{V}_6B\frac{R_2}{K_6+{R}_2}\end{array}\\ {}\begin{array}{c}\frac{d{R}_3}{dt}={V}_3{I}_3\frac{1-{R}_3}{K_3+1-{R}_3}-{V}_7O\frac{R_3}{K_7+{R}_3}\\ {}\frac{dO}{dt}={V}_4{R}_2\frac{1-O}{K_4+1-O}-{V}_8{R}_1\frac{O}{K_8+O}\end{array}\end{array}\right. $$

In this description, the total concentrations of enzymes are normalized, and the variables vary from 0 to 1. In the normal condition, the receptor nodes R_1_, R_2_, R_3_ sense the surroundings with a base signal *I*_1_ = *I*_2_ = *I*_3_ = 0.1. With the parameter values in Fig. 5, the system rests at its stable steady state (with *R*_1_ = 0.1179, *R*_2_ = 0.3431, *R*_3_ = 0.0024, *O* = 0.1690). When node R_1_ is stimulated with *I*_1_ ≥ 0.2 and *I*_2_ = *I*_3_ = 0.1, the system enters into the regime of self-sustained oscillation (Fig. 5a). Oscillations are generated due to the R_1_-R_3_-O negative feedback loop within the circuit. The step signal is thus interpreted by the system into stable limit cycle oscillation in the output node. As depicted in Fig. 5d, the network dynamics undergoes a subcritical Hopf bifurcation at *I*_1*C*_ = 0.25 when the strength of stimulus I_1_ is tuned as the control parameter.

For the second signal (*I*_2_ > 0.1, *I*_1_ = *I*_3_ = 0.1), the circuit’s output responds to the stimulus I_2_ transiently and returns to its pre-stimulated level. This transient activation results from the saturated regulations of R_3_ from both signal I_3_ and output node O, with *K*_3_ ≪ 1 − *R*_3_, *K*_7_ ≪ *R*_3_. In this case, Eq. 1c for R_3_ is simplified as,

2 $$ \frac{d{R}_3}{dt}\approx {V}_3{I}_3-{V}_7O $$

At steady state, the above equation leads to the output which is proportional to the amplitude of *I*_*3*_,

3 $$ {O}_{SS}=\frac{V_3{I}_3}{V_7} $$

The independence of *O*_*SS*_ on *I*_*2*_ results in a perfect adaptation. Thus, the necessary condition for adaptation under the second stimulus requires that the node R_3_ functions as a buffer node within the negative feedback loop in the circuit. Figure 5b shows the responses of the output variable when node R_2_ is triggered by *I*_*2*_ of different strengths. The steady state of the network dynamics as a function of the signal amplitude *I*_*2*_ is depicted in Fig. 5e.

When node R_3_ of the circuit receives the third stimulus (*I*_3_ > 0.1, *I*_1_ = *I*_2_ = 0.1) of different amplitudes, the output node is always activated (see Fig. 5c), and the equilibrium state of the output increases with I_3_ strength (Fig. 5f). Therefore, the three signal-encoding processes are mutually compatible and completed in the simplest and robust topology.

## Discussion

Biochemical reactions in cellular signaling processes form complex molecular networks analogous to electrical circuits. There has been a long tradition of theoretical studies that looked into modules of small biochemical networks and signaling motifs that are viewed as simple building blocks of complex networks. Proteins functioning as the transfer and processing of information can be linked into motifs of reoccurring biochemical circuits that perform specific tasks such as to amplify signal, integrate and store information et al. [51, 52]. Motifs of common features that may help formalize the mapping from biochemical reactions to pathway block diagrams in signaling processes have been previously examined [53, 54]. In signaling and transcription regulation networks, the main classes of motifs that can carry out specific dynamic functions such as bistability, adaptation, oscillations, excitable pulse and et al. have been reviewed in references such as [55] and [2]. Compared to previous studies that considered mainly mono-functional network motifs, we here report a systematic approach for extracting multi-functional motifs, i.e., depending on different external signals, the simple motifs can output dynamic responses of oscillation, transient activation, and sustained activation respectively.

The second novel point of this study lies in that competition between substrates for the same enzyme has been taken into account in our mathematical description of signaling circuits. Substrate competition that was usually omitted in previous theoretical studies can have pronounced effects. It has been reported that ultra-sensitivity can be generated through substrate competition between two sets of phosphorylation sites [56] or through negative cooperativity in which the binding of ligand to a multimeric receptor makes it more difficult for subsequent ligands to bind [57]. Competitive binding can lead to high-dose hook effect (also known as prozone effect) in reactions where one protein acts as a linker between parts of a complex [58]. In the interaction of Ca^2+^ and calcium binding proteins calmodulin (CaM), the competitive binding among CaM’s seven partners can tune the Ca^2+^/CaM binding frequency [59]. In this study, we demonstrate that competitive effects could add implicit interactions within the networks that can promote or hamper the anticipated behaviors, and even create new dynamics unexpected from the explicit network topologies. For simple network structures without explicit functional motifs, the additional hidden regulations within the competitive nodes, combined with the rest of the linkages in the networks, could form implicit functional motifs that foster the networks to achieve target functions. For complex network topologies with dense linkages within the network, such as in the bi-functional networks obtained by two-node merging, the competitive effects could produce more complicated connections within the circuits that can drastically influence the network dynamics.

The multiple signal-encoding networks we consider involve at least four nodes. It is time-consuming to exhaust computationally all possible network topologies. We have adopted a compromise approach to efficiently construct multi-functional networks by enumerating modules of three nodes that can achieve each sub-function and then combing them by node merging. As the exploration is not exhaustive, the tri-functional networks we obtained may have occupied only a part of all functional topologies. As demonstrated, we have constructed tri-functional networks with four to six nodes via module combinations. The functions of oscillation and adaptation in the tri-functional networks could share a negative feedback loop, as long as the circuit could be driven to oscillate by the first signal and a node within as a buffer node for adaptation in the presence of the second signal. Alternatively, the function of adaptation could also be achieved via approaches such as the negative feedback loop with a buffer node (M_2_) in networks N_1_, N_2_, or N_3_. The third function of sustained activation, it requires at least one activation regulation directly or indirectly from node R_3_ to the output node O.

Compared to the functional networks we designed, the real signal-encoding network of Msn2 in *Saccharomyces cerevisiae* is much more complex [45–50]. A recent report that integrated experiments and modelling revealed that, the stimulus-dependent Msn2 dynamics is controlled by coupled feedback loops [60]. At the core of this Msn2 pathway is the Ras–cAMP–PKA negative feedback loop which is crucial in shaping Msn2 dynamics. It is coupled to the signal-dependent mutual inhibition between protein kinase PKA and the yeast AMP-activated protein kinase Snf1. The negative feedback regulating PKA activity at the core of Msn2 pathway is abundant as sub-module in the functional networks that we identified here. As can be seen in Fig. 3 and Fig. 4, the negative feedback appears in forms of activator-inhibitor, delayed negative feedback, and repressilator. In the networks we found, coupled negative and positive feedback loops are not apparent. But when implicit interactions due to substrate competing are taken into account, such as those demonstrated in Fig. 2g, Fig. 2h, and in Fig. 3d, Fig. 3e, Fig. 3f, coupled negative and positive feedbacks are abundantly found as sub-modules in the networks identified here.

In p53 signaling pathway, the information of hundreds of cellular stress stimuli is encoded through the single node of p53 protein, and stimulus-specific p53 dynamics then triggers distinct cellular outcomes. While the real p53 signaling pathway is very complex, modelling studies [61] revealed that the flexibility of p53 activity is rooted in the core p53-Mdm2 negative feedback loop in the pathway: the transcription factor p53 activates the expression of Mdm2, and Mdm2 in turn inhibits p53 by either turning down its transcription or triggering its degradation. In the multifunctional networks that we find here, the negative feedback loop featuring the p53 pathway is also a fundamental sub-module. Negative feedback loops involving the output node are ubiquitous in bi- and tri- functional circuits we find here. In single cell study of p53 dynamics [17], γ-radiation and UV light cause DNA double strand breaks (DSBs) and single-stranded DNA (ssDNA) damage, respectively. It was uncovered that the mechanism of stimulus-specific response is relevant to two core coupled negative feedback loops. The upstream kinases ATM responding to DSBs and ATR responding to ssDNA both relay the damage signal to p53. This activates p53-Mdm2 and p53-Wip1 negative feedback loops. The core topology of two coupled negative feedback loops in p53 pathway in response to DNA damages is closely related to some of the networks that we identify here. As depicted in Fig. 3a, two coupled negative feedback loops are characteristic of the combined a_3_ + b_2_ and a_4_ + b_2_ networks, and apparently feature the combined networks in Fig. 3d and Fig. 3e. Although the tri-functional networks we constructed are simple and omit the real aspects of dynamic control, they could still be helpful for understanding the mechanism of real signal-encoding processes. Furthermore, the networks we obtained and the method we adopted could also be used in synthetic biology to construct multiple functional dynamic circuits for practical purposes.

## Conclusions

The three signal-processing functions are compatible in a well-designed multi-functional network, in which the output dynamics is switched between oscillation, adaptation, and sustained activation by changing the input node for the signals, with no need to change the internal links in the networks. Competition between substrates for limited enzymes plays as a framework that renders implicit interactions between the nodes that are not explicitly linked. The substrate competition brings virtual links between nodes in the network and can create new implicit motifs that are seemingly nonfunctional.

## Methods

We aim to search for networks capable of encoding the identity of distinct stimuli into the dynamics of the circuit’s output as a whole. The dynamics of oscillation, adaptation (transient activation) and sustained activation are the three target input-output responses considered. We assume that the functional network has three independent receptors each receiving a respective stimulus signal. Under normal conditions, all input signals are set as a low baseline, and the functional network remains in the resting state. When facing one of the three stimuli, the input signal is represented by a step function (Fig. 1a). The information about the stimulus is processed through the unknown components of the networks (Fig. 1b), and then the identity of the stimulus is encoded in the dynamics of the circuit’s output. The target dynamic outputs are illustrated in Fig. 1c as oscillations (F_1_), transient activation (F_2_), and sustained activation (F_3_). We intend to find the unknown networks in Fig. 1b that generate the target output when each receptor node receives its stimulus from an external signal.

In our calculations, the networks we consider are limited to enzymatic regulatory interactions. Each node in the network represents an enzymatic protein with a fixed total concentration (normalized to 1) and can be interconverted between its active and inactive forms by other enzymes that are active. Two types of links are considered. The arrow in the network denotes activation of the target enzyme, that is, the enzyme catalytically transforms its substrate from the inactive form to active form. The blunt-headed arrow indicates catalytic inactivation of the active substrate. In our model, we assume that nodes in the network can possibly be auto-activated or auto-inhibited. If a node in the network has no activation or deactivation from other nodes, it is assumed to be regulated by basally available enzymes in the background.

To take into account the competition effects among the enzymes that compete to bind the same target enzyme [42], we model the network dynamics with modified Michaelis-Menten rate equations. The dynamic variables represent the concentrations of enzymes (normalized to 1) in their active forms. For example, when enzyme R_1_ in the network converts both the enzymes M_1_ and O_1_ to active states M^*^_1_ and O^*^_1_ (Fig. 1d), the activation rate (RA) of M_1_ and O_1_ take the following forms (see additional file 1 for the full dynamical equations):

4 $$ {\displaystyle \begin{array}{l}{RAofM}_1={V}_1{R}_1\frac{\frac{1-{M}_1}{K_1}}{1+\frac{1-{M}_1}{K_1}+\frac{1-{O}_1}{K_2}}\\ {}{RAofO}_1={V}_2{R}_1\frac{\frac{1-{O}_1}{K_2}}{1+\frac{1-{M}_1}{K_1}+\frac{1-{O}_1}{K_2}}\end{array}} $$

where *R*_*1*_, *M*_*1*_ and *O*_*1*_ are the concentrations of active enzymes R^*^_1_, M^*^_1_ and O^*^_1_, *K*_*1*_ and *K*_2_ denote the Michaelis-Menten constants, and *V*_*1*_ and *V*_2_ denote the regulation rate constants. Due to competition between enzymes M_1_ and O_1_ for binding enzyme R^*^_1_, an increase in the concentration of O^*^_1_ and therefore a decrease in inactive O_1_ (due to the constant total concentration) would increase the concentration of enzymes available for the inactive M_1_. This leads to an increased rate of M^*^_1_ production and vice versa. The situation is similar when enzyme R^*^_1_ deactivates both active enzymes M^*^_1_ and O^*^_1_. In these two cases, the competition effect results in implicit positive interactions between the two nodes as illustrated in Fig. 1d and Fig. 1e (note the dashed arrows in the circuits). Figure 1f illustrates the situation when enzyme R^*^_1_ activates M_1_ but deactivates O^*^_1_, resulting in implicit negative interactions of suppression. These competitive effects inducing implicit positive or negative interactions in enzymatic circuits have commonly been omitted in most theoretical or experimental models.

For a concrete demonstration of the competition effect between substrates, Fig. 1g and Fig. 1h demonstrate with a simple three-node network the distinct temporal behaviors generated with modified Michaelis-Menten equations and the normal Michaelis-Menten equations, respectively. With the same set of parameter values and same initial conditions, the description that takes into account competitive bindings depicts self-sustained oscillations (Fig. 1g) while a stationary steady state is generated when the normal Michaelis-Menten mechanism is adopted (Fig. 1h). The ordinary differential equations for the circuit in Fig. 1g and Fig.1 h are listed in additional file 1. One sees that the competition effect generates implicit mutual activation interactions as demonstrated with dashed links in Fig. 1g and sustains a completely different dynamical behavior. From the circuit that is added with two more edges, the oscillations not expected in the original topology are supported due to that a negative feedback loop forms with the aid of the implicit interactions coming from the competition effects. The competition effect could thus play significant roles in the topology of functional circuits and need to be considered in the design principles of multiple signal-encoding functional networks.

We adopt the method of module combination to construct the multi-functional networks. The scenario consists of three steps. First, we search separately for functional network topologies capable of oscillation and adaptation by enumerating all possible networks with three nodes. Typical and simple functional networks, i.e., those topologies that have less links and are relatively more robust, are then selected to form module pools for these two signal-encoding sub-functions. The combined network topologies are then constructed from the two sub-functional pools by merging nodes. Those that are capable of achieving both oscillation and adaptation in response to external stimuli are selected as bi-functional circuits. The final topologies for our target multiple signal-encoding functions are completed by adding to the bi-functional circuits an extra node that directly activates the output node upon receiving the signal, or alternatively by adopting an existing regulatory node for input of the third signal that directly or indirectly activates the output node. Ultimately, we obtain tri-functional networks having four to six nodes.

The modified Michaelis-Menten equations for network dynamics are integrated numerically. The code is written in computer C language with Visual Studio 2008. The ODE solver *rkf45* of Runge-Kutta algorithm is chosen. The scripts are available upon request. Rate constant values ranging from 0.1 to 10 are considered. The Michaelis-Menten constant changes in the range 10^−3^~10. In order to determine computationally whether oscillations emerge out or not, the variance of network output variable in a time window is monitored after the transient behavior dies out. An apparently not zero variance indicates oscillations arise. The oscillations with both amplitude and oscillation period larger than 0.1 are considered in this study. To numerically determine adaptation, the transient peak value O_peak_ and asymptotic end value O_end_ of the output variable are monitored and calculate their differences from the initial value O_ini_. The numerical criteria for adaptation are that *O*_*peak*_ − *O*_*ini*_ > 0.2 and ∣*O*_*end*_ − *O*_*ini*_ ∣  < 0.1 are both satisfied. Sustained activation is determined by the criterion *O*_*end*_ − *O*_*ini*_ > 0.2.

In order to analyze topological properties of three-node networks with oscillations and adaptation, we perform clustering analyses for the functional networks as in [39]. In a three-node network, there are totally nine possible links because there are at most three links from each node to other nodes and to itself. Values of 1, − 1, and 0 are assigned for links of activation, inhibition, and no regulation, respectively. A network topology can be therefore described with a digital number of length nine. The topological difference between two networks having three nodes can then be measured by calculating the hamming pair-wise distance from the digital numbers. The clustering analyses of the functional networks is performed by using *clustergram* in Matlab. The clustering for bi-functional networks is similarly analyzed.

The original c code files used in our computation for enumeration of sub-functions (Additional file 2), and merging of functional modules (Additional files 3, 4, 5) as well as a model in SBML format (Additional file 6) for the typical network (N5 in Fig. 4c) can be found in Additional files.

## Additional files

Additional file 1: The appendix for the equations and parameters used in Fig.1(d-f), and module selection for the function of sustained activation, as well as the analysis of bi-functional mechanism for the hybrid motif of a3 + b1. (DOCX 165 kb)

Additional file 2: The original codes used in our computation for enumeration of sub-functions. (ZIP 2318 kb)

Additional file 3: The original codes used in our computation for one-node merging of functional modules. (ZIP 1608 kb)

Additional file 4: The original codes used for two-node merging of functional modules. (ZIP 1018 kb)

Additional file 5: The original codes used for three-node merging of functional modules. (ZIP 1162 kb)

Additional file 6: The SBML format model for the network N5 in Fig. 4c. (XML 9 kb)
