# Supplementary material for: Constructing network topologies for multiple signal-encoding functions
Source: BMC Syst Biol. 2019 Jan 11;13:6. doi: 10.1186/s12918-018-0676-5 (PMC6330498; doi:10.1186/s12918-018-0676-5)
Supplement: Supplementary file 1 — The appendix for the equations and parameters used in Fig.1(d-f), and module selection for the function of sustained activation, as well as the analysis of bi-functional mechanism for the hybrid motif of a3 + b1. (DOCX 165 kb) [file 12918_2018_676_MOESM1_ESM.docx]

## Appendix

**The equations and parameters used in Fig.1(d-f).**

In our model, there is background activation or deactivation on the node in case it has no activation or deactivation from other nodes. In Fig. 1d, enzyme R_1_ can convert both the substrates of M_1_ and O_1_ from their inactive to active states, and the rate equations for the active enzymes M^*^_1_ and O^*^_1_ take the following forms:

$\frac{dM_{1}}{dt}=V_{1}R_{1}\frac{\frac{1-M_{1}}{K_{1}}}{1+\frac{1-M_{1}}{K_{1}}+\frac{1-O_{1}}{K_{2}}} -V_{3}B_{3}\frac{M_{1}}{M_{1}+K_{3}}$

$\frac{dO_{1}}{dt}=V_{2}R_{1}\frac{\frac{1-O_{1}}{K_{1}}}{1+\frac{1-M_{1}}{K_{1}}+\frac{1-O_{1}}{K_{2}}} -V_{4}B_{4}\frac{O_{1}}{O_{1}+K_{4}}$

with parameters $R_{1}=B_{3}=B_{4}=1,V_{1}=V_{2}=V_{3}=V_{4}=1,K_{1}=0.1,K_{2}=0.2,K_{3}=K_{4}=1.$

In Fig. 1e, enzyme $R_{1}$ converts both the substrates of M_1_ and O_1_ from their active to inactive states; the dynamic equations are

$\frac{dM_{1}}{dt}=V_{1}B_{1}\frac{{1-M}_{1}}{{1-M}_{1}+K_{1}}-V_{3}R_{1}\frac{\frac{M_{1}}{K_{3}}}{1+\frac{M_{1}}{K_{3}}+\frac{O_{1}}{K_{4}}}$

$\frac{dO_{1}}{dt}=V_{2}B_{2}\frac{{1-O}_{1}}{{1-O}_{1}+K_{2}}-V_{4}R_{1}\frac{\frac{O_{1}}{K_{4}}}{1+\frac{M_{1}}{K_{3}}+\frac{O_{1}}{K_{4}}}$

with parameters $R_{1}=B_{1}=B_{2}=1,V_{1}=V_{2}=V_{3}=V_{4}=1,K_{1}=K_{2}=1,K_{3}=0.1,K_{4}=0.2.$

In Fig. 1f, enzyme $R_{1}$ converts the substrate M_1_ (O_1_) from its inactive (active) to active (inactive) state, and the equations are

$\frac{dM_{1}}{dt}=V_{1}R_{1}\frac{\frac{{1-M}_{1}}{K_{1}}}{1+\frac{{1-M}_{1}}{K_{1}}+\frac{O_{1}}{K_{4}}}-V_{3}B_{3}\frac{M_{1}}{M_{1}+K_{3}}$

$\frac{dO_{1}}{dt}=V_{2}B_{2}\frac{{1-O}_{1}}{{1-O}_{1}+K_{2}}-V_{4}R_{1}\frac{\frac{O_{1}}{K_{4}}}{1+\frac{{1-M}_{1}}{K_{1}}+\frac{O_{1}}{K_{4}}}$

with parameters $R_{1}=B_{2}=B_{3}=1,V_{1}=V_{2}=V_{3}=V_{4}=1,K_{2}=K_{3}=1,K_{1}=0.1,K_{4}=0.2.$

For the circuit in Fig 1g, the equations that take into account the implicit interactions (substrate competitive binding) take the form:

${\frac{dR_{1}}{dt}=V}_{1}I_{1}\frac{1-R_{1}}{K_{1}+1-R_{1}}-V_{4}O_{1}\frac{R_{1}}{K_{4}+R_{1}}$,

$\frac{dM_{1}}{dt}=V_{2}R_{1}\frac{\frac{1-M_{1}}{K_{2}}}{1+\frac{{1-O}_{1}}{K_{3}}+\frac{1-M_{1}}{K_{2}}}-V_{5}B_{1}\frac{M_{1}}{K_{5}+M_{1}}$,

$\frac{dO_{1}}{dt}=V_{3}R_{1}\frac{\frac{{1-O}_{1}}{K_{3}}}{1+\frac{{1-O}_{1}}{K_{3}}+\frac{1-M_{1}}{K_{2}}}-V_{6}B_{2}\frac{O_{1}}{K_{6}+O_{1}}$,

with parameters $V_{1}=0.2784$, $K_{1}=0.0021$, $V_{2}=0.2727$, $K_{2}=0.0016$,$V_{3}=2.9737$, $K_{3}=0.2602$, $V_{4}=1.9645$, $K_{4}=0.0596$,$V_{5}=0.5364$, $K_{5}=0.7396$,$V_{6}=0.6166$, $K_{1}=0.0082$,$B_{1}=0.5$, $B_{2}=0.5$, $I_{1}=0.5$, with initial condition [0.1, 0,1, 0.1].

For the circuit in Fig 1h, the equations that omit the implicit interactions are as follows,

${\frac{dR_{1}}{dt}=V}_{1}I_{1}\frac{1-R_{1}}{K_{1}+1-R_{1}}-V_{4}O_{1}\frac{R_{1}}{K_{4}+{RR}_{1}}$,

$\frac{dM_{1}}{dt}=V_{2}R_{1}\frac{1-M_{1}}{K_{2}+1-M_{1}}-V_{5}B_{1}\frac{M_{1}}{K_{5}+M_{1}}$,

$\frac{dO_{1}}{dt}=V_{3}R_{1}\frac{{1-O}_{1}}{{K_{3}+1-O}_{1}}-V_{6}B_{2}\frac{O_{1}}{K_{6}+O_{1}}$,

with parameters $V_{1}=0.2784$, $K_{1}=0.0021$, $V_{2}=0.2727$, $K_{2}=0.0016$,$V_{3}=2.9737$, $K_{3}=0.2602$, $V_{4}=1.9645$, $K_{4}=0.0596$,$V_{5}=0.5364$, $K_{5}=0.7396$,$V_{6}=0.6166$, $K_{1}=0.0082$,$B_{1}=0.5$, $B_{2}=0.5$, $I_{1}=0.5$, with initial condition [0.1, 0,1, 0.1].

**Module selection for the function of sustained activation.**

Positive regulation from the third input node R_3_ to the circuit’s output O is necessary for achieving the sustained activation response. Thus, for simplicity we selected the simplest activation structure ($R_{3}\longrightarrow O$) for the F_3_ pool.

**Fig.S1. Clustering of robust networks (Q_3_>1000) for F3.** There are 55 distinct robust three-node network topologies for F3. The common functional motif is shown at right.

**The analysis of bi-functional mechanism for the hybrid motif of a3 + b1.**


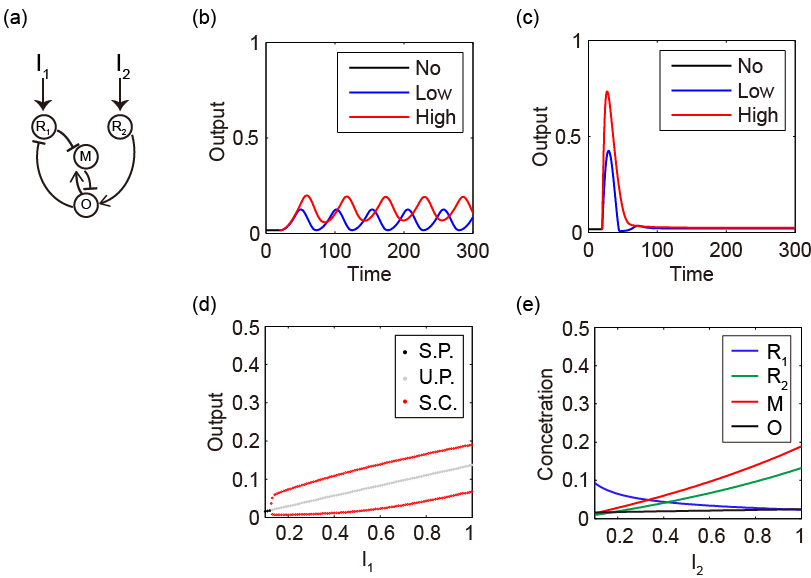
**Fig.S2. Analysis for the simplest bi-functional network containing the hybrid motif of a3 + b1.** (a) Network topology of the a3 + b1 network. (b) Dynamics simulation for F1. (c) Dynamics simulation for F2. (d) Bifurcation diagram for F1. (e) Stable equilibrium across the range of I_2_. The concentrations of all nodes are normalized. The unit of time is arbitrary. The circuit is in the normal state (receiving no stimuli, I_1_ = I_2_ = 0.1) during the time interval between 0 and 20. At the time of 20 in figure (b), I_1_ suddenly increases to 0.5 (low stimulus) or 1 (high stimulus), and then the output of the circuit turns to generating oscillation. At the time of 20 in figure (c), I_2_ suddenly increases to 0.5 (low stimulus) or 1 (high stimulus), and then the output of the circuit behaves like adaptation. Both in figure (b) and (c), the black lines denote the normal state, the blue lines denote the low stimulus state and the red lines denote the high stimulus state. In figure (d), the black and grey dots denote stable and unstable fixed points of the output node, respectively. And the red dots in figure (d) represent the maximum and minimum position of the stable limit cycle of the output node. The value of parameters in the circuit is as list: $V_{1}=0.8454$,$V_{2}=1.3544$,$V_{3}=0.1492$,$V_{4}=6.0559$,$V_{5}=6.5584$,$V_{6}=6.4083$,$V_{7}=0.2060$,$V_{8}=1.4963$,$K_{1}=0.0015$,$K_{2}=0.1876$,$K_{3}=9.8888$,$K_{4}=2.0216$,$K_{5}=0.0194$,$K_{6}=0.2486$,$K_{7}=6.6258$,$K_{8}=0.0021, B=0.5$.

The rate equations for the network in Fig.s2 take the following forms,

$$\left\{ \begin{matrix} \begin{matrix} {\frac{dR_{1}}{dt}=V}_{1}I_{1}\frac{1-R_{1}}{K_{1}+1-R_{1}}-V_{5}O\frac{\frac{R_{1}}{K_{5}}}{1+\frac{R_{1}}{K_{5}}+\frac{1-M}{K_{3}}} \\ \frac{dR_{2}}{dt}=V_{2}I_{2}\frac{1-R_{2}}{K_{2}+1-R_{2}}-V_{6}B\frac{R_{2}}{K_{6}+R_{2}} \end{matrix} \\ \begin{matrix} \frac{dM}{dt}=V_{3}O\frac{\frac{1-M}{K_{3}}}{1+\frac{R_{1}}{K_{5}}+\frac{1-M}{K_{3}}}-V_{7}R_{1}\frac{M}{K_{7}+M} \\ \frac{dO}{dt}=V_{4}R_{2}\frac{1-O}{K_{4}+1-O}-V_{8}M\frac{O}{K_{8}+O} \end{matrix} \end{matrix} \right.$$

The equations of the circuit have been normalized, thus the concentration range of all nodes is between 0 and 1. Under no stimulating condition (*I_1_ = I_2_* = 0.1), the circuit rest at the stable steady state ($R_{1}=0.09$,$R_{2}=0.01$,$M=0.01,O=0.02$). When the circuit receives the first stimulus ($I_{1}>0.1,I_{2}=0.1$) , the circuit undergoes a supercritical bifurcation ($I_{1C}=0.13$) and then turns to a stable limit cycle oscillation. The negative feedback loop within the circuit is the necessary condition for generating oscillation.

When the circuit receives the second stimulus ($I_{2}>0.1,I_{1}=0.1$), the circuit’s output responds to the stimulus transiently and returns to pre-stimulated level. When the regulation from I_1_ on R_1_ and the regulation from O on R1 are both nearly saturation ( $K_{1}\ll1-R_{1}$,$K_{5}\ll R_{1}$) , and the node O is more easily to interact with the R1 than M ($K_{5}\ll K_{3}$), the rate equation for B can be approximated by the following:

$${\frac{dR_{1}}{dt}\approx V}_{1}I_{1}-V_{5}O$$

The steady solution of the above equation is:

$$O_{SS}=\frac{V_{1}I_{1}}{V_{5}}$$

Thus, the necessary condition for performing adaptation under the second stimulus requires that the node R_1_ behaves as a buffer node within the negative feedback loop in the circuit.

If we remove the positive regulation from O to M in the above circuit, the node M needs an additional enzyme in the background to activate the node M. The new network could also perform the two functions under appropriate parameters. However, the robustness (Q_1,2_) is less than the former network (7 < 14).

**Q(F_3_|F_1,2_) reflects the robustness of the tri-functional network.**

The relationship between Q(F_3_|F_1,2_) and the probability of finding an appropriate parameter set for a network to perform the three signal-processing functions in a defined parameter space is as follows:

$$P\left( F_{1,2,3} \right)=P\left( F_{1,2} \right)\times\left( {F_{3}|F}_{1,2} \right)=\frac{Q(F_{1,2})}{10000}\times\frac{Q({F_{3}|F}_{1,2})}{1000\times Q(F_{1,2})}=\frac{Q({F_{3}|F}_{1,2})}{{10}^{7}}$$

$P\left( F_{1,2,3} \right)$ and $P\left( F_{1,2} \right)$ denote, respectively, the probability of finding an appropriate parameter set for a network to perform the three and the first two signal-processing functions in a defined parameter space. $P\left( {F_{3}|F}_{1,2} \right)$ denotes the conditional probability of finding an appropriate parameter set for a bi-functional network to perform the third signal-processing function in a defined parameter space. $Q({F_{3}|F}_{1,2})$ is proportional to $P\left( F_{1,2,3} \right)$, and thus the value of $Q({F_{3}|F}_{1,2})$ reflects the robustness of the tri-functional network.
